# Cryo-EM structures of tau filaments from Alzheimer’s disease with PET ligand APN-1607

**DOI:** 10.1007/s00401-021-02294-3

**Published:** 2021-03-16

**Authors:** Yang Shi, Alexey G. Murzin, Benjamin Falcon, Alexander Epstein, Jonathan Machin, Paul Tempest, Kathy L. Newell, Ruben Vidal, Holly J. Garringer, Naruhiko Sahara, Makoto Higuchi, Bernardino Ghetti, Ming-Kuei Jang, Sjors H. W. Scheres, Michel Goedert

**Affiliations:** 1 MRC Laboratory of Molecular Biology, Cambridge, UK; 2 APRINOIA Therapeutics, Taipei, Taiwan; 3 Department of Pathology and Laboratory Medicine, Indiana University School of Medicine, Indianapolis, IN 46202 USA; 4 National Institute of Radiological Sciences, National Institutes for Quantum and Radiological Science and Technology, Chiba, 263-8555 Japan

**Keywords:** Positron emission tomography, Alzheimer’s disease, Posterior cortical atrophy, Primary age-related tauopathy, Immunopurification, Electron cryo-microscopy

## Abstract

Tau and Aβ assemblies of Alzheimer’s disease (AD) can be visualized in living subjects using positron emission tomography (PET). Tau assemblies comprise paired helical and straight filaments (PHFs and SFs). APN-1607 (PM-PBB3) is a recently described PET ligand for AD and other tau proteinopathies. Since it is not known where in the tau folds PET ligands bind, we used electron cryo-microscopy (cryo-EM) to determine the binding sites of APN-1607 in the Alzheimer fold. We identified two major sites in the β-helix of PHFs and SFs and a third major site in the C-shaped cavity of SFs. In addition, we report that tau filaments from posterior cortical atrophy (PCA) and primary age-related tauopathy (PART) are identical to those from AD. In support, fluorescence labelling showed binding of APN-1607 to intraneuronal inclusions in AD, PART and PCA. Knowledge of the binding modes of APN-1607 to tau filaments may lead to the development of new ligands with increased specificity and binding activity. We show that cryo-EM can be used to identify the binding sites of small molecules in amyloid filaments.

**Supplementary Information:**

The online version contains supplementary material available at 10.1007/s00401-021-02294-3.

## Introduction

Assembly of a small number of soluble proteins into insoluble amyloid filaments underlies the majority of age-related neurodegenerative diseases [20]. Tau is the most commonly affected of these proteins. It assembles into filaments in a number of diseases, including Alzheimer’s disease (AD), chronic traumatic encephalopathy (CTE), progressive supranuclear palsy (PSP), globular glial tauopathy (GGT), corticobasal degeneration (CBD) and Pick’s disease (PiD). Six tau isoforms are expressed in the adult human brain. Three isoforms have three microtubule-binding repeats, whereas the other three have four repeats each. All six tau isoforms assemble in AD and CTE, but only four-repeat tau forms inclusions in PSP, GGT and CBD; PiD is a three-repeat tau proteinopathy.

AD is defined by the presence of abundant intraneuronal tau inclusions and extracellular Aβ deposits [41]. Cognitive impairment and neurodegeneration correlate better with the number and distribution of tau inclusions than with Aβ assemblies. Although silver staining, amyloid dyes and antibodies that label tau assemblies in post mortem brains have been available for many years, PET ligands that detect tau inclusions in living subjects have only recently been developed. Cross-sectional studies have shown that lower brain volumes are more strongly associated with tau-PET than Aβ-PET signals [8]. A prospective longitudinal study has indicated that the levels of tau-PET signal predict rates of brain atrophy [29], in agreement with neuropathological studies [4].

First-generation tau PET probes can be divided into three major chemical groups, with ^18^F-THK5351 [22], ^18^F-flortaucipir [7] and ^11^C-PBB3 (pyridinyl-butandienyl-benzothiazole 3) [35] being representative compounds. ^11^C-PBB3 is the most promising for visualizing inclusions made of only 3R or 4R tau isoforms. However, rapid conversion into a metabolite results in low entry of unmetabolized ^11^C-PBB3 into the brain. Recently, it has been shown that second-generation tau PET probe ^18^F-APN-1607, also called ^18^F-PM-PBB3 (propanol modification of PBB3), a compound with high metabolic stability, labels brain regions with abundant tau inclusions in AD and PSP [26, 46]. Here we used cryo-EM structure determination to identify the binding sites of APN-1607 in PHFs and SFs from AD.

## Materials and methods

### Clinical history and neuropathology

Tau filaments were extracted from the frontal cortex of neuropathologically confirmed cases of AD with sarkosyl (case 2 in reference [15]) or by affinity chromatography [28]. Occipital cortex from a previously described individual (case 18 in reference [15]) with PCA was used for tau filament extraction with sarkosyl. Tau filaments were extracted with sarkosyl from the entorhinal cortex or hippocampus of 3 previously undescribed individuals with PART. Case 1, with definite PART, was a male who died aged 88 following a 2-year history of progressive memory loss. In addition to tau changes, prominent large- and small-vessel cerebrovascular disease associated with white matter loss was observed; small, healed cerebral infarcts were present in the frontal cortex and basal ganglia. There was substantial atrophy of the visual system, consistent with a history of long-term blindness. This individual, who suffered numerous head injuries, was declared legally blind at age 21. Signs of a healed subdural haemorrhage were seen at autopsy. α-Synuclein and TDP-43 inclusions were not observed. Case 2, with definite PART, was a male who died aged 65 with cognitive impairment. There were signs of extensive small vessel disease; TDP-43 inclusions were present in nerve cells and glial cells, mostly in some subcortical regions. α-Synuclein inclusions were not detected. Case 3, with possible PART, was a female who died aged 88 following a 2-year history of progressive memory loss. α-Synuclein or TDP-43 inclusions were not observed. Additional findings are summarized in Table 1.

**Table 1:** Cases of Alzheimer’s disease, posterior cortical atrophy and primary age-related tauopathy used for sarkosyl extraction and cryo-EM

Diagnosis	Gender	Age at death (y)	Brain region	Tau stage	Aβ phase	Aβ angiopathy scale	*APOE* haplotypes
AD	F	82	Frontal cortex	VI	5	3	*ε*4*/ε*4
PCA	M	63	Occipital cortex	VI	5	3	*ε*4*/ε*4
PART1	M	88	Hippocampus	IV	0	0	*ε*2*/ε*3
PART2	M	65	Entorhinal cortex	I	0	0	*ε*3*/ε*3
PART3	F	88	Hippocampus	III	2	3	*ε*3*/ε*4

Alzheimer’s disease (AD) (case 2) and posterior cortical atrophy (PCA) (case 18) have been described [15]. Primary age-related tauopathy (PART) cases 1–3 have not been described before. There were no known disease-causing mutations in *MAPT*. PART cases 1–3 had a heterozygous T185S change in *TMEM106B* (gene encoding transmembrane protein 106B). PART case 1 had a heterozygous R62H change in *TREM2* (gene encoding triggering receptor expressed on myeloid cells 2) and a heterozygous N357S change in *TIA1* (gene encoding cytotoxic granule-associated RNA-binding protein). Whole-exome sequencing did not detect mutations known to cause AD, Parkinson’s disease, frontotemporal dementias or amyotrophic lateral sclerosis. PART case 1 was also heterozygous for mutations C195F and D1005V in *CRB1* (gene encoding crumbs homologue 1). Biallelic mutations in *CRB1* cause retinal disease [31], consistent with the development of blindness in this individual. His mother also suffered from retinal disease and was blind. Braak tau stages, Thal Aβ phases and Olichney Aβ angiopathy scales were determined according to references [4, 39, 47]

### Histology, immunohistochemistry and silver staining

Histology and immunohistochemistry were carried out as described [45]. Briefly, 8 μm paraffin-embedded tissue sections were treated for antigen retrieval, prior to overnight incubation with primary antibodies at room temperature. The sections were counterstained with haematoxylin/eosin and Luxol fast blue. Primary antibodies were: RD3 tau [10], anti-4R tau [12], AT8 [36], anti-phospho TDP-43 [23, 27], anti-Aβ 6F/3D [33] and anti-Aβ 4G8 [32] (Supplemental Fig. 1, online resource). Secondary antibodies were either ImmPRESS HRP anti-rabbit or anti-mouse IgG (Vector, Burlingame, CA), with 3,3′-diaminobenzidine used as chromogen. Some sections were silver-impregnated using the method of Gallyas-Braak [5].

### Fluorescence staining of tau deposits with APN-1607

Fluorescence staining of brain sections from cases of AD, PCA and PART with APN-1607 were done as described [40, 46]. Briefly, deparaffinized 6 μm sections were incubated in 50% ethanol containing 25 μM APN-1607 at room temperature for 30 min. They were rinsed in 50% ethanol for 5 min., washed twice in water for 3 min. and mounted in non-fluorescent media (Vectashield, Vector Laboratories). Fluorescence images were captured using a DM4000 microscope (Leica) equipped with a custom filter cube, as described [46].

### Whole-exome sequencing

Target enrichment made use of the SureSelectTX human all-exon library (V6, 58 megabase pairs; Agilent) and high-throughput sequencing was carried out using a HiSeq 4000 (sx75 base-pair paired-end configuration; Illumina). Bioinformatics analyses were performed as described [17].

### Extraction of tau filaments

Sarkosyl-insoluble material was extracted from hippocampus and cortex (frontal, occipital and entorhinal) [19]. Tissues were homogenized in 10 ml/g tissue of extraction buffer consisting of 10 mM Tris–HCl, pH 7.5, 10% sucrose, 0.8 M NaCl, 5 mM EDTA, 1 mM EGTA and a protease and phosphatase inhibitor (Thermo Fisher). Homogenates were spun at 20,000 g for 20 min. and supernatants retained. Pellets were homogenized in 5 ml/g extraction buffer and centrifuged at 20,000*g* for 20 min. Both supernatants were combined, brought to 1% sarkosyl and incubated for 60 min. at room temperature. Following a 60 min. centrifugation at 100,000*g*, pellets were resuspended in 250 μl/g extraction buffer and spun at 20,000*g* for 20 min. The resulting supernatants were centrifuged at 100,000 g for 1 h. For cryo-EM, pellets were resuspended in 25 μl/g tissue of 20 mM Tris–HCl, pH 7.5, 100 mM NaCl. Tau filaments were immunopurified from the frontal cortex of two individuals with neuropathologically confirmed AD (cases a and b), as described [28].

### Binding of APN-1607 to PHFs and SFs.

APN-1607 was synthesised as described [26]. A 10 mM solution was prepared using anhydrous dimethyl sulfoxide (DMSO, Thermo Fisher) and stored at − 20 °C. Tau filaments were sarkosyl-extracted from frontal cortex of an individual who had died with a neuropathologically confirmed diagnosis of AD (case 2 in reference [15]) and incubated with 100 μM APN-1607 in 20 mM Tris–HCl, pH 7.5, 100 mM NaCl, 1% DMSO, for 3 h at room temperature (25 μl/g tissue). The concentration of APN-1607 was 2.5 nmol/g tissue. Controls were incubated with buffer containing 1% DMSO. Given the photosensitivity of APN-1607, these experiments were carried out in the dark, except for a yellow light source with a wavelength greater than 525 nm.

### Electron cryo-microscopy

Following a 1 min spin at 3000 g, 3 μl of supernatant were placed on glow-discharged holey carbon grids (Quantifoil Au R1.2/1.3, 300 mesh) and plunge-frozen in liquid ethane using an FEI Vitrobot Mark IV. The images of PART case 2 were acquired using a Falcon III detector in linear mode on a Glacios cryo-transmission electron microscope (Thermo Fisher) at 200 kV. All other images were acquired using a Gatan K2 summit detector in counting mode on a Titan Krios (Thermo Fisher) at 300 kV. A GIF quantum energy filter (Gatan) was used with a slit width of 20 eV to remove inelastically scattered electrons. Further details are given in Supplemental Fig.  2, online resource.

### Helical reconstruction

Movie frames were motion-corrected and dose-weighted using the motion correction implementation of RELION [51]. Aligned and non-dose weighted micrographs were used to estimate the contrast transfer function (CTF) by CTFFIND-4.1 [42]. Other image-processing steps were performed using helical reconstruction in RELION [24, 43]. Filaments were picked manually and extracted using an inter-box distance of 14.1 Å. For reference-free 2D classification, segments with a box size comprising an entire helical crossover were downscaled by a factor of 2–4 to speed up calculations. Different types of filament were separated by reference-free 2D classifications and segments contributing to suboptimal 2D averages discarded. For immunopurified AD tau filaments, as well as sarkosyl-extracted tau filaments from PART cases 1 and 3, initial 3D models were constructed de novo from 2D class averages comprising an entire helical crossover using the relion_helix_inimodel2d program [43]. For the other datasets, EMD-0259 (PHF) and EMD-0260 (SF) (from AD case 2 in reference [15]) were used as the initial 3D models. Helical twists were estimated either by crossover distances from 2D class averages, or according to those reported [15]. Segments for 3D auto-refinement were then re-extracted using a box size comprising approximately 33% of the helical crossover, without downscaling. With an initial 3D model that was low-pass filtered to 10 Å, 3D auto-refinement was carried out for several rounds with optimization of helical twist and rise, after reconstructions had shown separation of β-strands along the helical axis. Since PHF protofilaments are related by an approximate 2_1_ screw symmetry [18], additional helical symmetry was applied from the initial 3D refinements. We then performed Bayesian polishing and CTF refinement, followed by 3D classification without further image alignment, to remove segments that yielded suboptimal 3D reconstructions. To further improve the structure of the β-helix region of SF protofilament 2 from PART case 3, we used focused 3D classification (with signal subtraction) of V337-S356 without further image alignment. Final reconstructions were sharpened using standard post-processing procedures in RELION [51]. Overall resolution estimates were calculated from the Fourier shell correlations (FCSs) at 0.143 between two independently refined half-maps, using phase-randomisation to correct for convolution effects of a generous, soft-edged solvent mask that extended to 20% of the height of the box. Using the relion_helix_toolbox program [43], helical symmetries were imposed on the post-processed maps, which were then used for model building and refinement. For further details, see Supplemental Figs. 2 and 3, online resource.

### Model building and refinement

6HRE for PHFs [15], 6HRF for SFs [15] and 6NWP for CTE type I filaments [16] were used as initial references. Models containing five β-sheet rungs were refined in real-space by PHENIX [1] using local symmetry to keep all rungs identical. For SFs from PART case 3, the models were refined against the map before focused 3D classification, followed by real-space refinement in COOT [13] of only the turns between β-strands 4 and 5 against the map after focused 3D classification. MolProbity [6] was used for model validation. To confirm the absence of overfitting, FSC curves between one half-map and the model, which was refined against the other half-map, were checked. Additional details are given in Supplemental Figs. 2 and 3, online resource.

### Quantitation of extra densities

For each dataset, 3 independent refinements were carried out by dividing the cryo-EM images 3 times into different half-sets in RELION. They used the same references and parameters, without optimisation of helical symmetry. Each reconstruction was post-processed, with a low-pass filter of 3.0 Å and a B-factor of − 70 Å^2^ for the PHF maps, and a low-pass filter of 3.55 Å and a B-factor of − 55 Å for the SF maps. Helical symmetry was applied to post-processed half-maps using the relion_helix_toolbox program [43]. The reconstructions with a box size of 256 pixels and a pixel size of 1.15 Å were up-sampled to 558 pixels, from which the central rung could be extracted in an integer number of pixels (i.e., 9 pixels) along the helical axis. The central 18 slices were used for analysis. Binary masks were then created for the different binding sites; a solvent region extending 29 pixels around the entire filaments; and for residues 349–370 (binding sites 1, 2a, 2b, 3, 4) or 318–340 (binding sites 5, 6a, 6b) of tau. Relative densities were calculated as follows:

$$ {\text{Relative density }}\left( {\text{binding site}} \right) = \left[ {{\text{Max}}\left( {\text{binding site}} \right) - {\text{Avg}}\left( {{\text{solvent}}} \right)} \right]:\left[ {{\text{Max}}\left( {{\text{protein}}} \right) - {\text{Avg}}\left( {{\text{solvent}}} \right)} \right], $$

where: Max(binding site) is the highest pixel value within the mask for the binding site; Avg(solvent) is the average pixel value in the solvent region; Max(protein) is the highest pixel value in the protein density for residues 349–370 and 318–340 of tau (using the numbering of the 441 amino acid isoform of human brain tau).

## Results and discussion

### Structures of tau filaments from Alzheimer’s disease with APN-1607

**Fig. 1:**
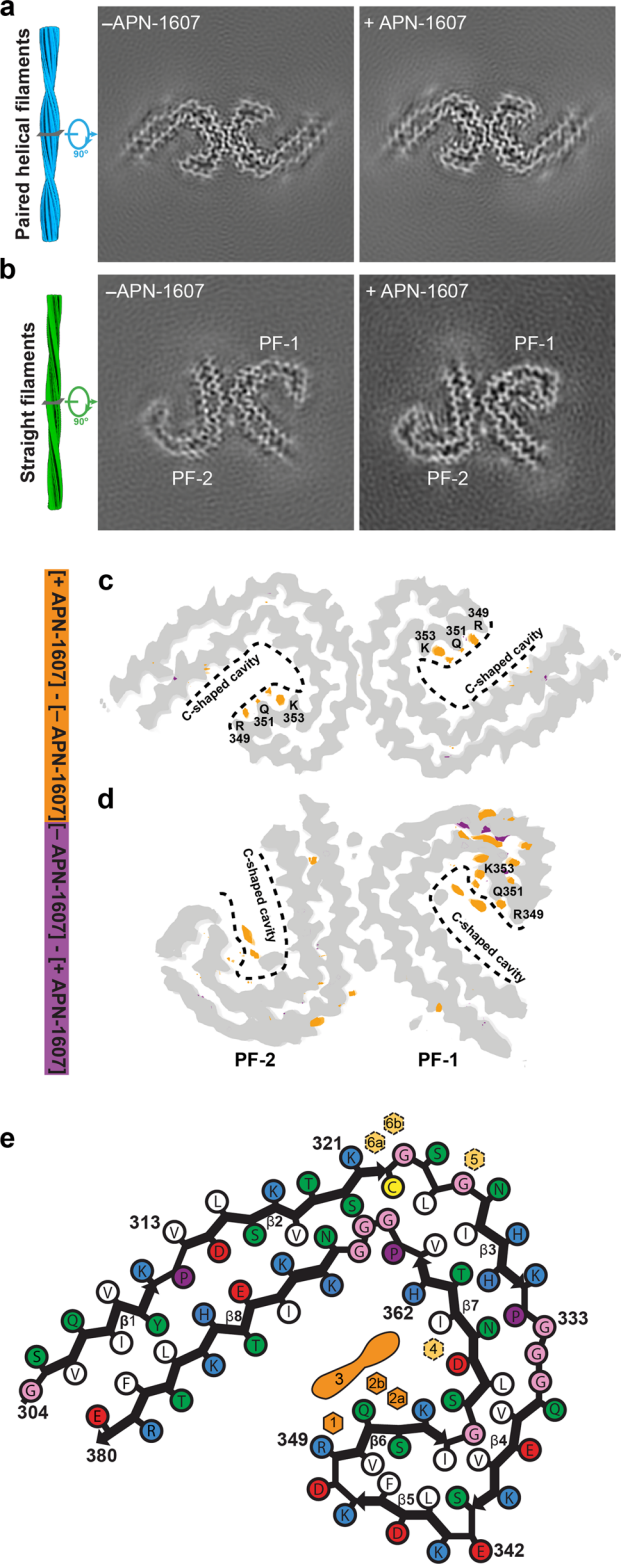
Cryo-EM maps of PHFs and SFs from Alzheimer’s disease with APN-1607. **a**, **b** Cryo-EM maps of tau filaments without (-APN-1607) and with (+APN-1607) PET ligand. **c**, **d**, Overlay of positive (orange) and negative (purple) difference maps and -APN1607 maps (grey). SF protofilaments 1 and 2 are labelled as PF-1 and PF-2. Amino acids R349, Q351 and K353 are indicated, with the C-shaped cavities outlined by stippled lines. The thresholds in the difference maps were 11 standard deviations for PHFs and 8 standard deviations for SFs. **e**, Alzheimer tau protofilament core with APN-1607 binding sites. Major binding sites 1, 2a, 2b and 3 are shown in orange; minor binding sites 4, 5, 6a and 6b are indicated in yellow

We added APN-1607 to sarkosyl-insoluble tau filaments from the frontal cortex of case 2 of AD (in reference [15]), prior to imaging by cryo-EM (+APN-1607). We used the PET ligand at 2.5 nmol/g. The *B*_max_ of ^18^F-PM-PBB3 for tau aggregates in AD frontal cortex has been reported to be 5.7 nmol/g [46]. The mean concentration of sarkosyl-insoluble tau is around 1 nmol/g cerebral cortex from AD [49]. The approximate molar APN-1607: tau ratio was therefore 2.5. As controls, we imaged tau filaments following the addition of only buffer (-APN-1607). By using helical reconstruction in RELION [24, 43], we determined the structures of PHFs to 3.0 Å (+APN-1607) and 2.8 Å (–APN-1607). There were fewer SFs, giving resolutions of 3.6 Å (+APN-1607) and 3.2 Å (–APN-1607). These maps enabled us to study additional densities that were well separated from those of tau (Fig. 1; Supplemental Fig. 3, online resource).

**Fig. 2:**
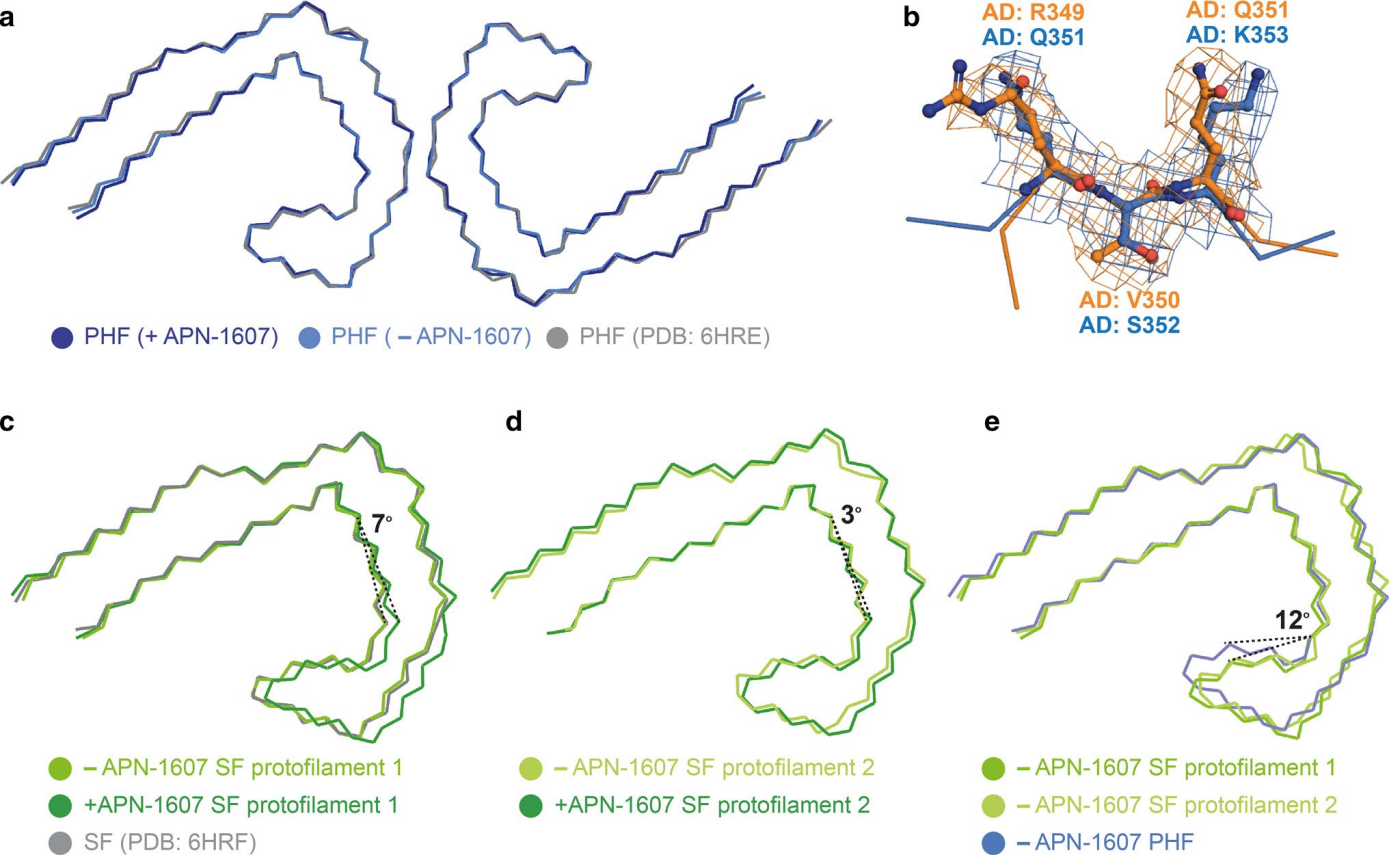
Alzheimer tau folds with and without APN-1607. **a**, Overlay of the structures of PHFs (+APN-1607; -APN-1607; PDB: 6HRE). **b** Overlay of R349-Q351 (binding site 1) and Q351-K353 (binding site 2) of PHFs. Flanking residues are shown as main-chain traces. **c**, **d**, Overlay of the structures of SF protofilaments 1 (**c**) and 2 (**d**) (+APN-1607; -APN-1607; PDB: 6HRF). **e**, Overlay of SF protofilaments 1 and 2, and the PHF protofilament (−APN-1607). The structures were aligned based on residues 363–380 of tau (in the numbering of the 441 amino acid isoform of human brain tau)

For PHFs, we observed additional densities in the + APN-1607 map compared to the –APN-1607 map (Fig. 1a, c, e), with the overall structure being unchanged (Fig. 2a). The root mean-square deviation between refined atomic coordinates in both maps was 0.4 Å. In the -APN-1607 map, densities in these grooves were weaker, approaching the intensity levels of noise features in the solvent region, suggesting that the extra densities in the +APN-1607 map corresponded to the PET ligand. In a difference map, these sites showed up as well-resolved peaks when displayed at a threshold of 11 standard deviations (Fig. 1c).

One additional density was present in the groove between the side chains of R349 and Q351 from β6 of the Alzheimer tau fold (binding site 1). Two additional densities were observed in the groove between the side chains of Q351 and K353 (binding sites 2a and 2b), possibly reflecting the width of this groove (Figs. 1, 2b). It is possible that side-to-side interactions between APN-1607 molecules at sites 2a and 2b, e.g. π-stacking between the π-electron-conjugated backbone of APN-1607, are required for binding at site 2b, and that APN-1607 may not be able to bind to site 2b when site 2a is empty. Despite a similar hydrophobic environment, the density at site 1 was weaker than at site 2a. These same grooves between the side chains that formed binding sites 1, 2a and 2b were previously identified by molecular docking among the predicted binding sites for multiple tau PET ligands [21, 38].

In SFs, protofilament 1 was better defined than protofilament 2. Nevertheless, we observed similar extra densities in both protofilaments at binding sites 1, 2a and 2b, as well as an additional elongated density within the C-shaped cavity (binding site 3) (Fig. 1b, d, e). When comparing the + APN-1607 and –APN-1607 maps, we observed a 7° difference in the orientation of β7 for protofilament 1 and a 3° difference for protofilament 2 (Fig. 2c, d). This suggests that the binding of APN-1607 induces a conformational change that enlarges the size of the C-shaped cavity in SFs. Even in the absence of APN-1607, this cavity is wider in SFs than in PHFs. Thus, an approximate 12° difference in the turn between β6 and β7 was observed when comparing PHFs and SFs in the –APN-1607 maps (Fig. 2e). When displaying the difference maps for SFs at a threshold of 8 standard deviations, all the binding sites showed up as well-resolved peaks and the conformational changes resulting from APN-1607 binding led to additional differences in SFs (Fig. 1d).

**Fig. 3:**
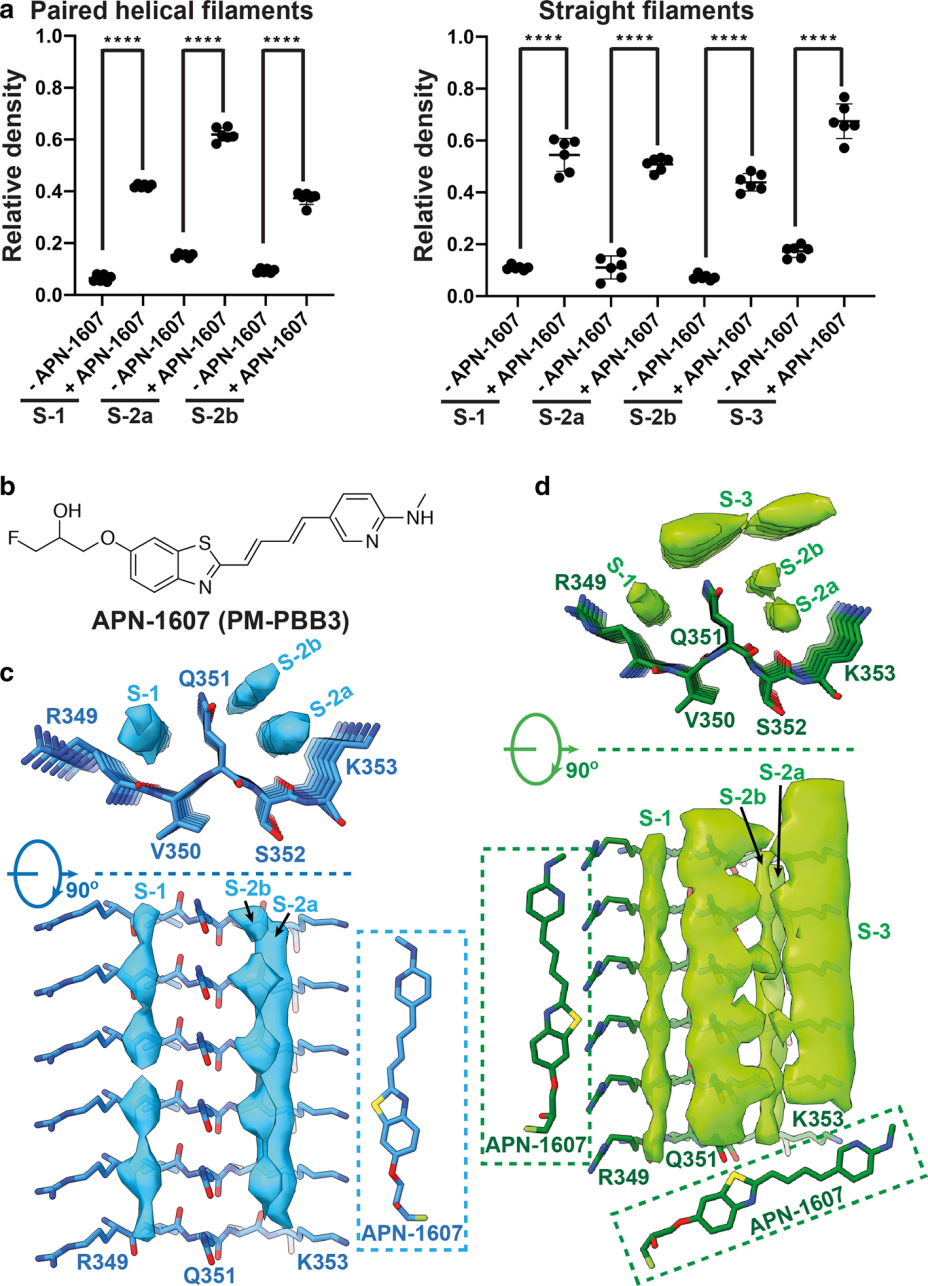
Binding of APN-1607 to PHFs and SFs. **a**, Relative densities at binding sites 1, 2a, 2b and 3 in cryo-EM maps of PHFs and SFs. Means, standard deviations and individual values of 6 half-set reconstructions are shown. Unpaired two-tailed t-test: ****p < 0.0001. **b**, Chemical structure of APN-1607 (PM-PBB3). **c**, **d**, Top views and side views of the extra densities in the APN-1607 binding sites of PHF (**c**) and SF (**d**) maps. The models of APN-1607 are shown near these extra densities at the same scale

To rule out that the weak densities at sites 1, 2a and 2b were caused by random noise, we performed 3 map refinements for each dataset, by dividing the cryo-EM images 3 times into different half-sets. We then quantified the densities at binding sites 1, 2a and 2b in each of the 6 half-set reconstructions relative to the densities of tau (see Methods). The additional densities at the various binding sites were weaker than the densities of tau. For PHFs, the additional densities were approximately 40% relative to the density of tau for site 1, 60% for site 2a and 40% for site 2b. For SFs, the relative densities were approximately 55%, 50% and 45%, respectively. In the absence of APN-1607, they were 5–15% in PHFs and SFs (Fig. 3a).

To exclude that sarkosyl binds to the same sites and interferes with APN-1607 binding, we determined the cryo-EM structures of tau filaments immunopurified from the frontal cortex of two neuropathologically confirmed cases of AD (cases a and b) (Supplemental Fig.4, online resource). For case (a), cryo-EM micrographs showed PHFs and SFs in a ratio of approximately 4:1. Using helical reconstruction in RELION [24, 43], we determined the structures of PHFs to 2.9 Å and SFs to 7.0 Å resolution (Supplemental Figs. 3b and 4a, online resource). For case (b), we observed mainly PHFs and determined their structure to 3.2 Å resolution (Supplemental Figs. 3b and 4b, online resource). We found no significant differences between sarkosyl-extracted and immunopurified filaments (Supplemental Fig. 4c, online resource). Immunopurified filaments also showed weak densities in both grooves. Therefore, we exclude sarkosyl as the reason for weak additional densities in the –APN-1607 structures. It is possible that molecules from the brain bind with low occupancy at these sites.

APN-1607 (Fig. 3b) fits into the elongated densities observed at binding sites 1, 2a and 2b, suggesting that it binds parallel to the long helical axis of amyloid filaments (Fig. 3c, d). A similar mode of binding has been reported for thioflavin-T, Congo red and luminescent conjugated oligothiophenes [25, 34, 44]. It remains to be seen if other tau PET ligands bind in the same grooves of the Alzheimer tau fold. Binding sites 1 and 2 have Q351 in common, which adopts an extended conformation. Although the orientation of its amide headgroup cannot be determined at the current resolution, it probably forms a hydrogen-bonded ladder along the filament axis, providing a rigid partition between sites 1 and 2. The positively charged headgroups of R349 and K353 are kept at a distance from Q351 by electrostatic interactions with the carboxylic groups of D348 and D358, creating predominantly hydrophobic grooves with polar edges as the binding sites. It follows that bound APN-1607 with a hydrophobicity log *p* value of 4.01 is stabilized mostly by hydrophobic interactions; occasional hydrogen bonds may form between its polar atoms and the side chains and/or carbonyl oxygens of tau. Additional densities are mostly featureless along the filament axis, suggesting that they do not obey helical symmetry. Therefore, the relative positions of individual APN-1607 molecules in the direction parallel to the helical axis cannot be determined from the cryo-EM maps. In addition, the blurring that results from the helical averaging in the reconstruction process complicates the interpretation of the relative densities in terms of the stoichiometry of ligand binding at the different sites.

The elongated shape of the additional density at binding site 3, with a density level of approximately 70% relative to that of tau (Fig. 3a, d), suggests that in SFs APN-1607 is able to bind in a direction nearly perpendicular to the helical axis, although the exact orientation and relative positions of individual molecules cannot be determined from the cryo-EM map. This site was not identified by molecular docking [21, 38]. At binding site 3, APN-1607 is probably stabilized on one side by hydrophobic interactions with APN-1607 molecules at binding sites 1 and 2b. On the other side, APN-1607 faces two additional densities of unknown nature. They have also been observed in the cryo-EM maps of PHFs and SFs from the frontal cortex of 4 cases of AD [15]. The finding that in PHFs there is less space between these additional densities and the β-helix at the tip of the C may explain why binding site 3 is not occupied. Weaker densities were also found in grooves other than those of binding sites 1 and 2. We refer to these minor binding sites as 4, 5, 6a and 6b (Fig. 1e; Supplemental Fig. 5 , online resource). In SFs, binding at sites 5, 6a and 6b was only seen in protofilament 2. Binding at site 6b was not observed in PHFs. A statistical analysis of the relative densities of all APN-1607 binding sites in PHFs and SFs from AD is given in Supplemental Fig. 6, online resource. The presence of an additional major APN-1607 binding site in SFs suggests that the ratio between PHFs and SFs could influence ligand binding, in particular the *B*_max_.

The tau protofilament folds of CTE, PiD and CBD differ from that of AD [14, 16, 18, 50]. Binding studies have suggested that APN-1607 may also be able to image tau inclusions of PiD and CBD [46]. It will be important to identify the binding sites of APN-1607 in tau filaments from these diseases. In the Pick fold, molecular docking has identified a potential binding site for APN-1607 in the groove between R349 and Q351 of tau [37, 46].

### Structures of tau filaments from posterior cortical atrophy and primary age-related tauopathy

PCA, a variant of AD, is characterized by an initial decline in visual processing, with the early presence of abundant tau inclusions and Aβ assemblies in the occipital cortex [3]. PART [9] has been described as a pathological continuum, ranging from localized entorhinal and hippocampal tau inclusions in individuals with normal or mildly impaired cognition to widespread tau inclusions in patients with tangle-only dementia (TD) [2, 9, 48]. It has also been proposed that PART is part of the events that lead to the presence of abundant plaques and tangles in neocortex, with a clinical picture of AD [11].

**Fig. 4:**
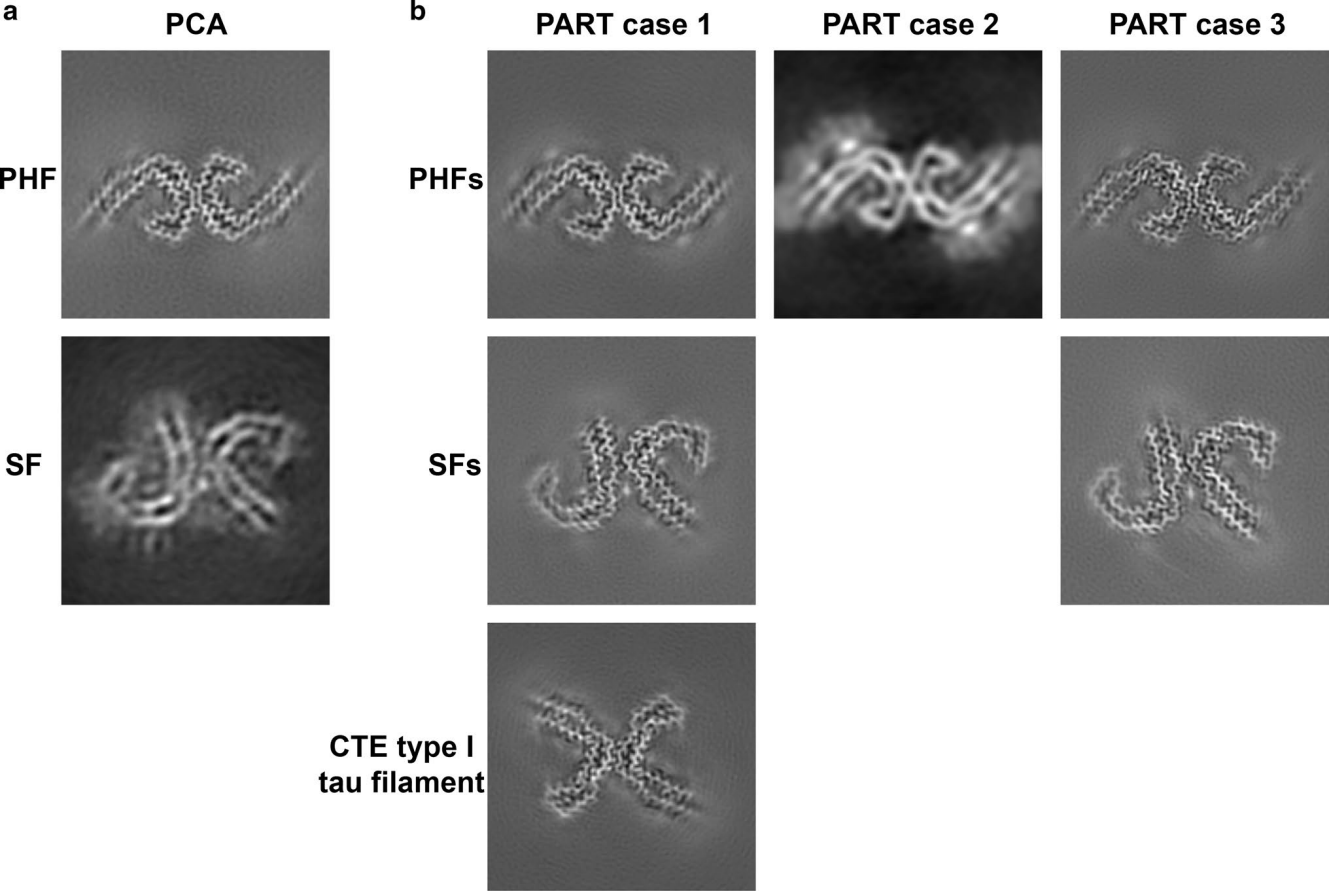
Cryo-EM maps of tau filaments from posterior cortical atrophy and primary age-related tauopathy. **a** PHF and SF from the occipital cortex of a case of PCA. **b** PHFs and SFs from the hippocampus of PART cases 1 and 3 and PHF from the entorhinal cortex of PART case 2; 13% of filaments from PART case 1 were CTE type I tau filaments

We determined cryo-EM structures of tau filaments from the occipital cortex of PCA (Fig. 4a). PHFs and SFs were present in a ratio of 9:1. Reconstructions calculated in RELION [24, 43] were identical to those determined previously from the frontal cortex of AD [15, 18]. The resolutions were 3.6 Å for PHFs and 5.2 Å for SFs (Fig. 4a; Supplemental Fig. 3b, online resource). We also imaged two cases of definite PART and one case of possible PART by cryo-EM and immunohistochemistry (Fig. 4b; Supplemental Figs. 7–10, online resource). Silver-positive tangles were present in the hippocampus of cases 1 and 3 and the entorhinal cortex of case 2. Sarkosyl-insoluble tau filaments from the hippocampus of PART case 1, who suffered from long-term blindness, had 26% PHFs and 61% SFs; 13% were CTE type I filaments [16]. The presence of CTE type I filaments was consistent with a history of head trauma and the presence of tau inclusions around blood vessels in occipital lobe (Supplemental Fig. 7, online resource). PART case 2 had only PHFs, whereas PART case 3 had 42% PHFs and 58% SFs. TDP-43 inclusions were present in subcortical brain regions of PART case 2 (Supplemental Fig. 8, online resource). Aβ deposits were observed in cortical regions of PART case 3, but not PART case 1 or 2 (Supplemental Fig. 10, online resource). We determined the structures of PHFs to resolutions of: 3.2 Å (PART case 1), 5.8 Å (PART case 2) and 2.8 Å (PART case 3). For SFs, the resolutions were: 2.8 Å (PART case 1) and 2.7 Å (PART case 3).

**Fig. 5:**
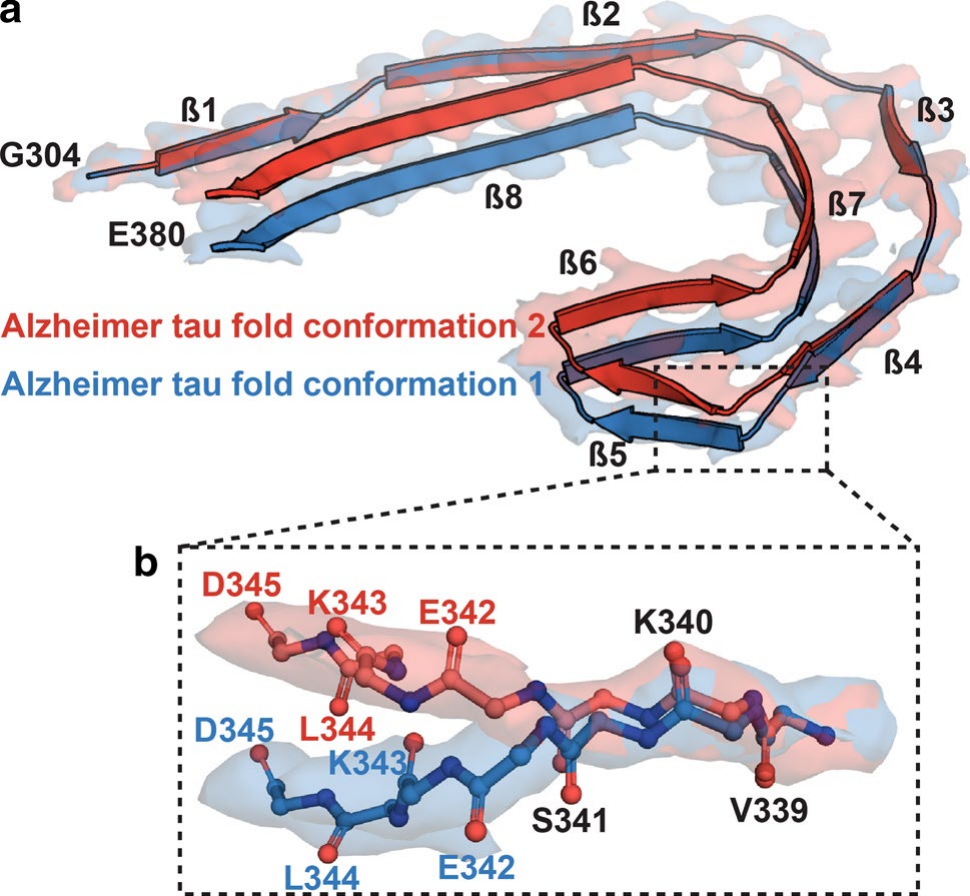
Two conformations of Alzheimer tau fold in SF protofilament 2 from PART case 3. **a** Overlay of two conformations of the Alzheimer tau fold from SF protofilament 2. The structures were aligned based on amino acids 304–340 of tau (in the numbering of the 441 amino acid isoform of human brain tau). **b** Close-up view showing that the difference between conformations was a flip of the peptide groups between E342 and its flanking residues, S341 and K343

In previous maps [15, 18], the density of SF protofilament 2 was not of sufficient quality to allow unambiguous chain tracing in the direction along the helical axis, in particular the β-helix region. Given the overall appearance of the density in the plane perpendicular to the helical axis, where the interactions between amino acid side chains shaped the Alzheimer tau fold, we assumed that the main chain would adopt the same fold in both protofilaments. At 2.7 Å, the cryo-EM map from PART case 3 has the highest known resolution for a SF (Figs. 4b and 5). We carried out further 3D classification that showed two distinct conformations (Fig. 5a). They were distinguished by flips of the peptide bonds between E342 and its flanking residues, S341 and K343 in conformation 2, which led the turn between strands 4 and 5 of the β-helix to connect to strands in adjacent rungs, with the rest of the structure being unchanged (Fig. 5b). Conformation 1 was identical to the tau fold of the PHF and SF protofilament 1. Conformation 2 was more similar to the CTE tau fold. The mixing of conformations suggests that in a substantial proportion of SFs, protofilament 2 can give rise to a subtype of the Alzheimer tau fold that is distinct from the fold of the PHF and from that of the SF protofilament 1. This could play a role in the asymmetric packing of SF protofilaments.

These findings establish that the Alzheimer tau fold can form in the absence of Aβ deposits and support the observation that homogenates from the brains of patients with AD and PART had similar seeding activities [30].

### Staining of tau inclusions from Alzheimer’s disease, posterior cortical atrophy and primary age-related tauopathy with APN-1607

**Fig. 6:**
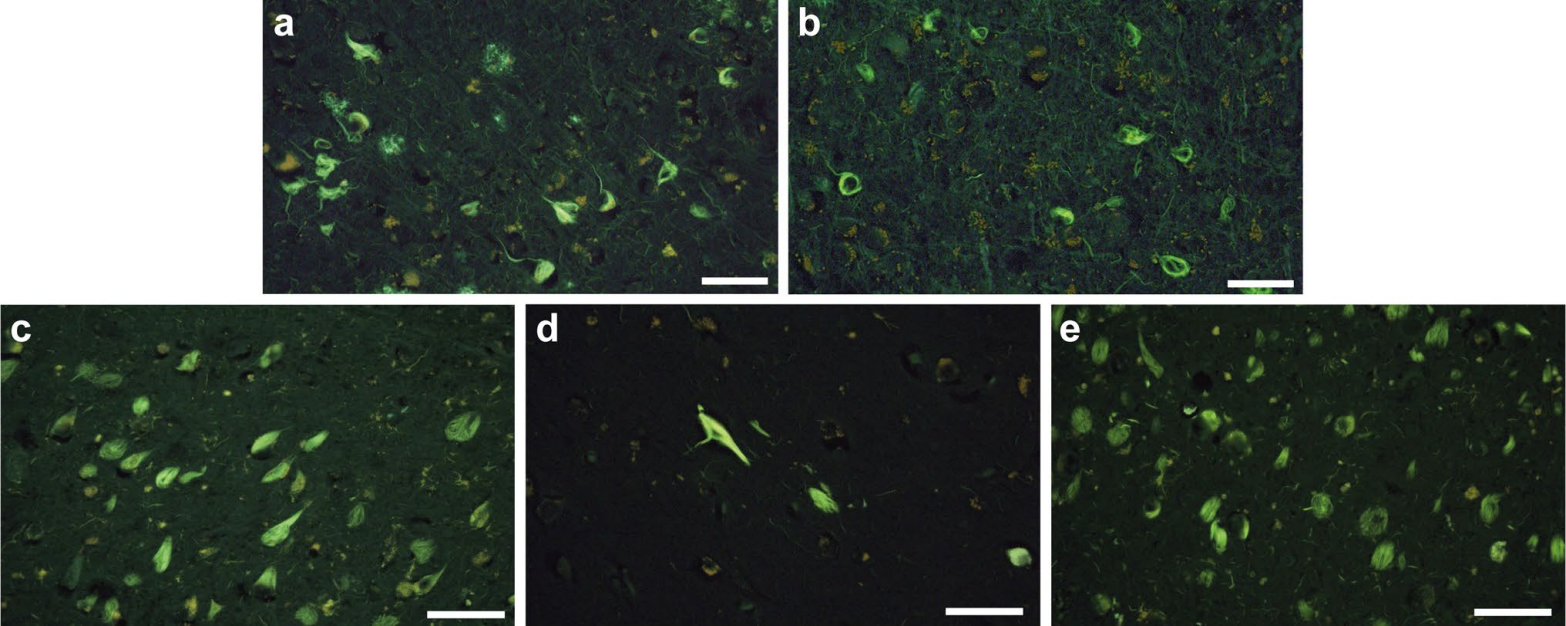
Staining of tau inclusions with fluorescent APN-1607. Sections from the frontal cortex of AD, occipital cortex of PCA, hippocampus of PART cases 1 and 3, and entorhinal cortex of PART case 2 were stained with APN-1607. The same cases of the disease were used as for cryo-EM. **a** AD; **b** PCA; **c** PART case 1; **d** PART case 2; **e** PART case 3. Scale bar, 50 μm

Since APN-1607 is fluorescent, its reactivity with tau inclusions can be investigated in brain sections [46]. APN-1607 labelled numerous nerve cell body inclusions in tissue sections from the cases of AD (Fig. 6a), PCA (Fig. 6b) and PART (Fig. 6c–e) used for cryo-EM, providing a link, not only with AD, but also with PCA and PART. Some neuritic plaques were also labelled (Fig. 6a). Triple staining of brain sections with APN-1607, an antibody against phosphorylated tau and Gallyas-Braak silver staining has shown binding of APN-1607 to tau inclusions in AD [46]. In PART, the number of stained inclusions was proportional to the Braak stages. Thus, PART case 2, with tau pathology at Braak stage I, had the smallest number of fluorescent inclusions (Fig. 6d). It follows that APN-1607 may also be useful as a PET ligand for the tau inclusions of PCA and PART.

### Conclusion

Taken together, our findings show that APN-1607 binds in the β-helix region of PHFs and SFs. This work establishes the use of cryo-EM to study the binding of small molecule compounds to amyloid filaments, which will allow the design of new PET ligands with increased specificity and binding activity for a range of neurodegenerative diseases.

## Supplementary Information

Below is the link to the electronic supplementary material.

Supplementary file1 (PDF 8607 KB)

## Declarations

### Open Access

Cryo-EM maps have been deposited in the Electron Microscopy Data Bank (EMDB) under accession numbers EMD-12551 for +APN-1607 PHF, EMD-12552 for +APN-1607 SF, EMD-12553 for PHF of PCA case, EMD-12549 and EMD-12550 for PHF and SF of PART case 3, respectively. Refined atomic models have been deposited in the Protein Data Bank (PDB) under accession numbers 7NRV for + APN-1607 PHF, 7NRX for + APN-1607 SF, 7NRQ, 7NRS and 7NRT for PHF, SF conformation 1 and SF conformation 2 of PART case 3, respectively. Whole-exome sequencing data have been deposited in the National Institute on Aging Alzheimer’s Disease Data Storage Site (NIAGADS; https://www.niagads.org), under accession number NG00101.
