# Correction to: Cryo-EM structures of tau filaments from Alzheimer’s disease with PET ligand APN-1607

**DOI:** 10.1007/s00401-021-02303-5

**Published:** 2021-04-08

**Authors:** Yang Shi, Alexey G. Murzin, Benjamin Falcon, Alexander Epstein, Jonathan Machin, Paul Tempest, Kathy L. Newell, Ruben Vidal, Holly J. Garringer, Naruhiko Sahara, Makoto Higuchi, Bernardino Ghetti, Ming-Kuei Jang, Sjors H. W. Scheres, Michel Goedert

**Affiliations:** 1 MRC Laboratory of Molecular Biology, Cambridge, UK; 2 APRINOIA Therapeutics, Taipei, Taiwan; 3 Department of Pathology and Laboratory Medicine, Indiana University School of Medicine, Indianapolis, IN 46202 USA; 4 National Institute of Radiological Sciences, National Institutes for Quantum and Radiological Science and Technology, Chiba, 263-8555 Japan

## Correction to: Acta Neuropathologica 10.1007/s00401-021-02294-3

In the original publication, second author name in reference 30 was incorrectly published as Tredici KD instead of Del Tredici K.

The correct reference text should read as:

Kaufman SK, Del Tredici K, Thomas TL, Braak H, Diamond MI (2018) Tau seeding activity begins in the transentorhinal/entorhinal regions and anticipates phospho-tau pathology in Alzheimer’s disease and PART. Acta Neuropathol 136:57–67.

The original article has been corrected.

