# Supplementary material for: Cryo-EM structures of tau filaments from Alzheimer’s disease with PET ligand APN-1607
Source: Acta Neuropathol. 2021 Mar 16;141(5):697–708. doi: 10.1007/s00401-021-02294-3 (PMC8043864; doi:10.1007/s00401-021-02294-3)
Supplement: Supplementary file 1 — Supplementary file1 (PDF 8607 KB) [file 401_2021_2294_MOESM1_ESM.pdf]

Cryo-EM structures of tau filaments from Alzheimer's disease with PET ligand APN-1607

Yang Shi[1], Alexey G. Murzin[1], Benjamin Falcon[1], Alexander Epstein[1], Jonathan Machin[1], Paul Tempest[2], Kathy L. Newell[3], Ruben Vidal[3], Holly J. Garringer[3], Naruhiko Sahara[4], Makoto Higuchi[4], Bernardino Ghetti[3], Ming-Kuei Jang[2], Sjors H.W. Scheres[1]*† and Michel Goedert[1]*†

[1]MRC Laboratory of Molecular Biology, Cambridge, UK.

[2]APRINOIA Therapeutics, Taipei, Taiwan.

[3]Department of Pathology and Laboratory Medicine, Indiana University School of Medicine, Indianapolis, IN 46202, USA

[4]National Institute of Radiological Sciences, National Institutes for Quantum and Radiological Science and Technology, Chiba 263-8555, Japan

* Corresponding authors. Email: mg@mrc-lmb.cam.ac.uk (M.G.); scheres@mrc-lmb.cam.ac.uk (S.H.W.S.). Telephone number: +44 (0)1223 267056 (M.G.); +44 (0)1223 267061 (S.H.W.S.)

† These authors jointly supervised this work

Name	Epitope	Supplier	Species	Type	IHC dilution	Validation
RD3 tau	Tau 209-224	Millipore	Mouse	Monoclonal	1:3,000	[10]
anti-4R tau	Tau 275-291 (N279D)	Cosmo Bio	Rabbit	Polyclonal	1:400	[12]
AT8	Tau pS202/pT205	Thermo Fisher	Mouse	Monoclonal	1:300	[36]
anti-phospho TDP-43	TDP-43 pS409/pS410	Cosmo Bio	Mouse	Monoclonal	1:800	[23, 27]
6F/3D	β-amyloid 8-17	Agilent Dako	Mouse	Monoclonal	1:100	[33]
4G8	β-amyloid 17-24	BioLegend	Mouse	Monoclonal	1:1,000	[32]

Supplemental figure 1

List of primary antibodies

	+APN-1607 PHF: EMD-12551 SF: EMD-12552	–APN-1607	Immunopurification AD case a	Immunopurification AD case b	PCA PHF:EMD-12553	PART 1	PART 2	PART 3 PHF: EMD-12549 SF: EMD-12550
**Data collection and processing**								
Electron microscope type	Titan Krios	Titan Krios	Titan Krios	Titan Krios	Titan Krios	Titan Krios	Glacios	Titan Krios
Nominal magnification	105,000	105,000	105,000	105,000	105,000	105,000	92,000	105,000
Voltage (kV)	300	300	300	300	300	300	200	300
Detector	K2 Summit	K2 Summit	K2 Summit	K2 Summit	K2 Summit	K2 Summit	Falcon III	K2 Summit
Electron exposure (e–/Å^2)	50.6	54.8	51.2	51.31	52.96	52.0	44.8	58.0
Defocus range (μm)	-1.0 to -3.0	-1.4 to -2.8	-1.0 to -3.5	-1.0 to -3.0	-1.0 to -3.5	-1.5 to -3.0	-1.5 to -3.5	-1.3 to -3.2
Pixel size (Å)	1.15	1.15	1.055	1.15	1.15	1.15	1.58	0.82
Micrographs (no.)	2,169	589	2,838	5,740	1,931	834	1,286	4,854
Initial particle images (no.)	749,433	428,100	144,568	174,851	790,297	540,264	73,551	1,810,902
Final particle images (no.)	PHFs: 277,058 SFs: 20,075	PHFs: 326,318 SFs: 26,837	PHFs: 112,172 SFs: 31,663	PHFs: 80,734	PHFs: 95,375 SFs: 41,971	PHFs: 53,878 SFs: 222,145 CTE type I: 62,027	PHFs: 31,541	PHFs: 425,943 SFs: 475,025 SFs1: 31,311 SFs2: 14,886
Map resolution (Å; FSC=0.143)	PHF: 3.00 SF: 3.55	PHF: 2.80 SF: 3.17	PHF: 2.91 SF: 6.95	PHF: 3.17	PHF: 3.55 SF: 5.16	PHF: 3.17 SF: 2.83 CTE type I: 3.13	PHF: 5.80	PHF: 2.76 SF: 2.68 SF1: 2.97 SF2: 3.26
Helical rise (Å)	PHF: 2.37 SF: 4.75	PHF: 2.38 SF: 4.77	PHF: 2.38 SF: 4.60	PHF: 2.37	PHF: 2.37 SF: 4.74	PHF: 2.38 SF: 4.77 CTE type I: 2.37	PHF: 2.375	PHF: 2.37 SF: 4.75 SF1: 4.76 SF2: 4.77
Helical twist (°)	PHF: 179.45 SF: -1.02	PHF: 179.45 SF: -1.09	PHF: 179.42 SF: -1.05	PHF: 179.46	PHF: 179.44 SF: -1.05	PHF: 179.46 SF: -1.07 CTE type I: 179.44	PHF: 179.45	PHF:179.44 SF: -1.08 SF1: -1.02 SF2: -1.08

	+APN-1607 (PHF) PDB: 7NRV	+APN-1607 (SF) PDB: 7NRX	PART 3 (PHF) PDB: 7NRQ	PART 3 (SF, conformation 1) PDB: 7NRS	PART 3 (SF, conformation 2) PDB: 7NRT
**Refinement**					
Initial model used	6HRE	6HRF	6HRE	6HRF	6HRF
Model resolution (Å; FSC=0.5)	3.10	3.39	2.69	2.69	2.63
Map sharpening *B* factor (Å)	-89.20	-76.13	-67.21	-50.40	-50.40
Model composition					
Non-hydrogen atoms	5870	5870	5870	5870	5870
Protein residues	770	770	770	770	770
Protein B factor (Å)	50.52	75.09	10.58	57.72	57.49
R.m.s. deviations					
Bond lengths (Å)	0.007	0.010	0.006	0.009	0.009
Bond angles (°)	1.145	1.466	1.115	1.079	1.089
Validation					
MolProbity score	1.55	1.86	1.70	1.61	1.50
Clashscore	3.52	6.37	5.03	6.04	4.53
Poor rotamers (%)	0	0	0	0	0
Ramachandran plot					
Favored (%)	94.00	91.33	93.20	96.00	96.00
Allowed (%)	100	100	100	100	100
Disallowed (%)	0	0	0	0	0

Supplemental figure 2

Cryo-EM data collection, model refinement and validation statistics

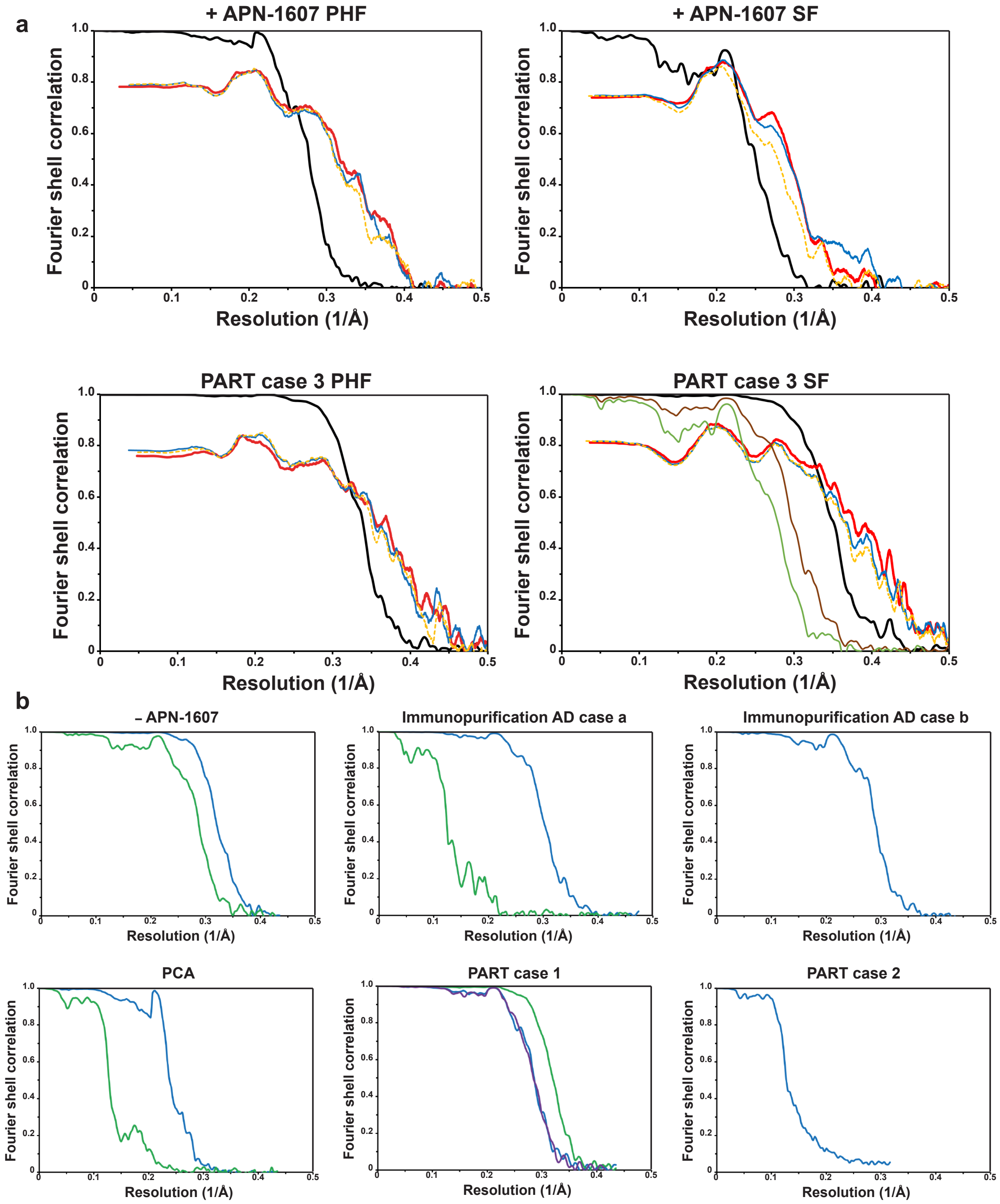

Supplemental figure 3

Cryo-EM maps and model comparisons

(a), PHF (+APN-1607) and SF (+APN-1607) from the frontal cortex of Alzheimer's disease case 2 (in reference [15]), as well as PHF and SF from the hippocampus of PART case 3. Fourier shell correlation (FSC) curves of two independently refined half-maps

(black line); FSC curves of final cryo-EM reconstruction and refined atomic model (red); FSC curves of the first half-map and the atomic model refined against this map (blue); FSC curves of the second half-map and the atomic model refined against the first half-map (yellow dashes). For SFs from PART case 3, in addition to the FSC curves for the consensus map and model, the FSC curves for two independently refined half-maps for conformation 1 and 2, which were identified after focused classification, are shown in brown and green, respectively.

(b), FSC curves of two independently refined half-maps of PHFs (blue), SFs (green) and CTE type I filaments (purple) from the frontal cortex of two cases of AD (immunopurified), occipital cortex from an individual with PCA, hippocampus of PART case 1 and entorhinal cortex of PART case 2

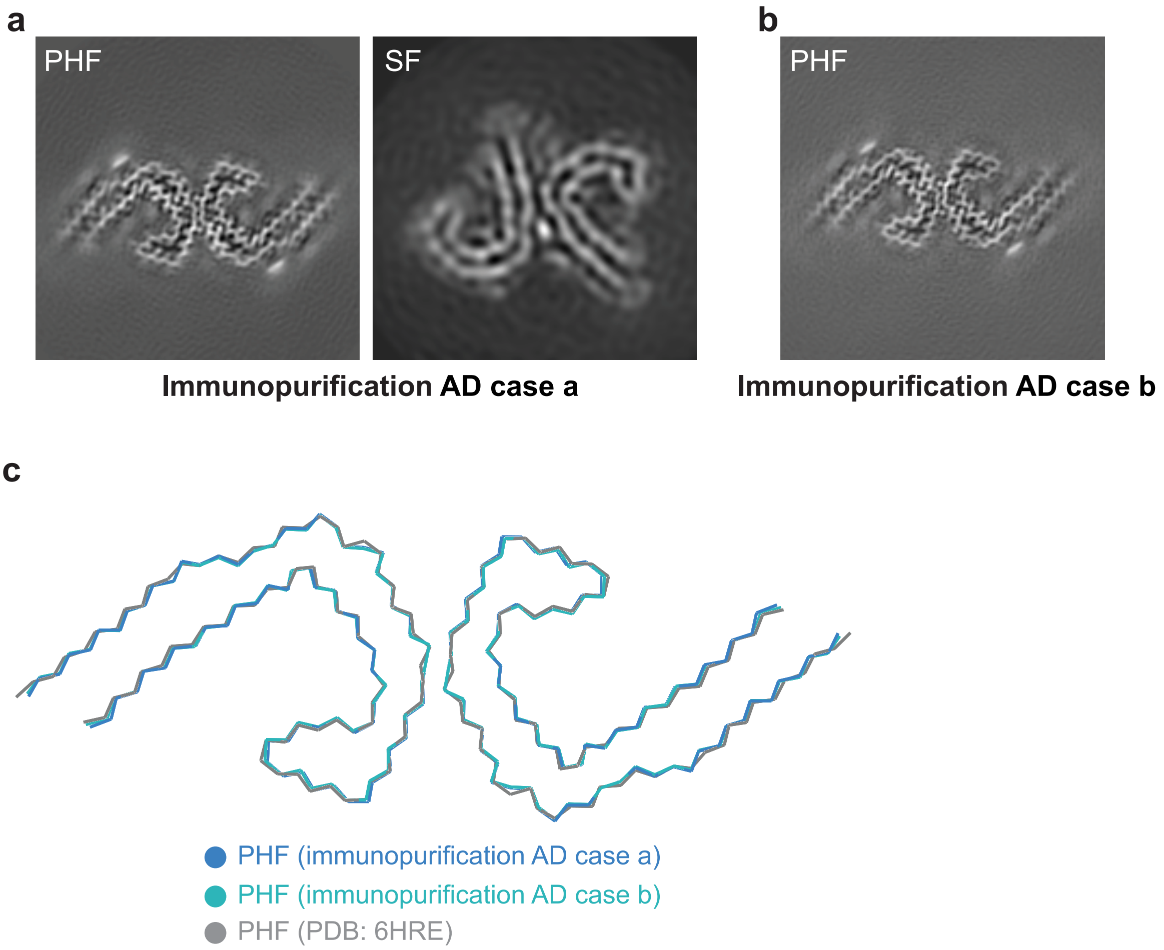

Supplemental figure 4

Cryo-EM maps of immunopurified PHFs and SFs from Alzheimer's disease (cases a and b)

(a), PHF and SF from the frontal cortex of case a.

(b), PHF from the frontal cortex of case b.

(c), Overlay of the structures of immunopurified PHFs (cases a and b) and sarkosyl-extracted PHF (PDB: 6HRE).

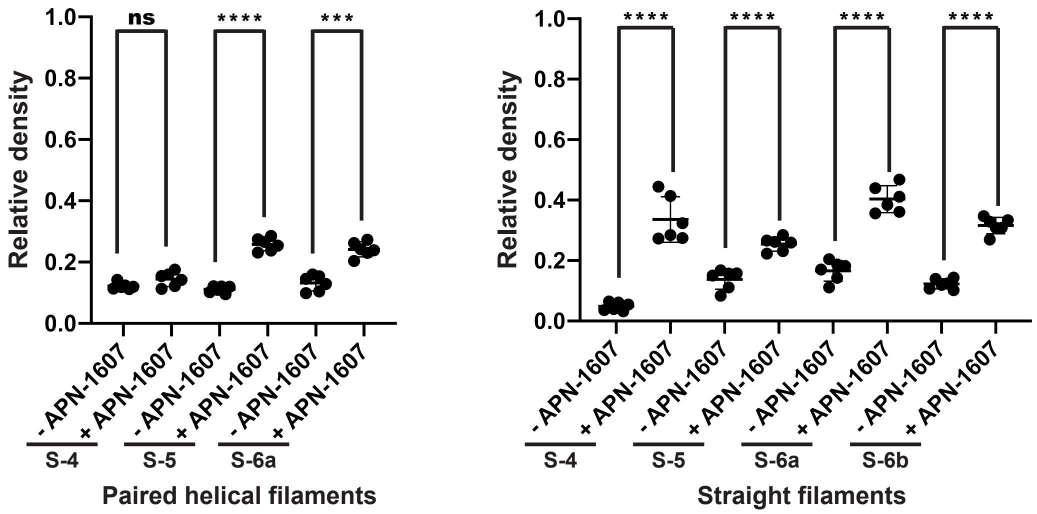

Supplemental figure 5

Relative densities of minor APN-1607 sites

Relative densities of minor sites (4, 5, 6a and 6b) in PHF and SF maps. Means, standard deviations and individual values of 6 half-set reconstructions are shown. The PHF maps were negative for S-6b. Unpaired two-tailed t test: ****$p<0.0001$; ns, not significant.

Binding site	Ligand	Mean	S.D.	n	Unpaired two-tailed t test			F test
					P	T	Degrees of Freedom	P
**Paired helical filaments**								
1	–APN-1607	0.0647	0.0136	6	6.0904E-14	57.55	10	0.1536
	+APN-1607	0.4207	0.0068					
2a	–APN-1607	0.1527	0.0078	6	9.7447E-13	43.57	10	0.0222
	+APN-1607	0.6196	0.0251					
2b	–APN-1607	0.0936	0.0082	6	1.2632E-10	26.68	10	0.0326
	+APN-1607	0.3738	0.0244					
4	–APN-1607	0.1218	0.0108	6	0.0585	2.135	10	0.1058
	+APN-1607	0.1445	0.0238					
5	–APN-1607	0.1114	0.0108	6	3.2385E-08	15.12	10	0.1686
	+APN-1607	0.2583	0.0212					
6a	–APN-1607	0.1317	0.0258	6	1.8674E-05	7.587	10	0.9054
	+APN-1607	0.2416	0.0244					
**Straight filaments**								
1	–APN-1607	0.1100	0.0087	6	1.3231E-08	16.59	10	0.0005
	+APN-1607	0.5445	0.0636					
2a	–APN-1607	0.1106	0.0445	6	3.8457E-09	18.84	10	0.2753
	+APN-1607	0.5088	0.0264					
2b	–APN-1607	0.0727	0.0105	6	1.9969E-10	25.47	10	0.0235
	+APN-1607	0.4390	0.0336					
3	–APN-1607	0.1731	0.0238	6	8.8147E-09	17.30	10	0.0405
	+APN-1607	0.6744	0.0669					
4	–APN-1607	0.0484	0.0137	6	3.3846E-06	9.203	10	0.0020
	+APN-1607	0.3359	0.0753					
5	–APN-1607	0.1381	0.0330	6	3.4777E-05	7.055	10	0.4606
	+APN-1607	0.2545	0.0233					
6a	–APN-1607	0.1664	0.0339	6	1.1484E-06	10.36	10	0.5620
	+APN-1607	0.4035	0.0446					
6b	–APN-1607	0.1229	0.0169	6	3.9774E-08	14.80	10	0.3197
	+APN-1607	0.3160	0.0271					

Supplemental figure 6

Statistical analysis of relative densities at binding sites

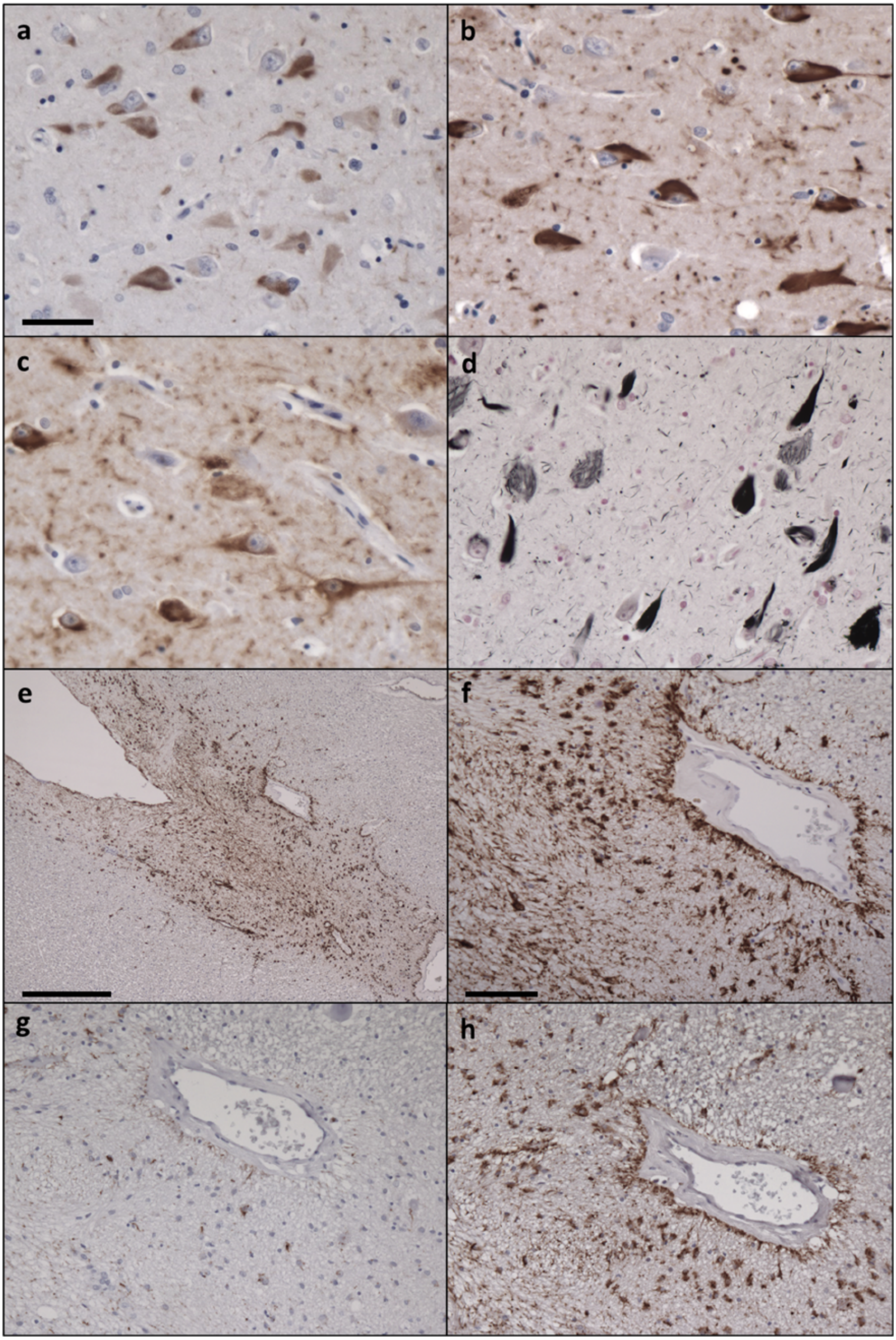
a
b
c
d
e
f
g
h

Supplemental figure 7

Tau staining of hippocampus and occipital cortex from PART case 1

Immunohistochemical staining of sections from hippocampus (a-d) and occipital cortex (e-h) using anti-tau antibodies RD3 (a,g), anti-4R (b,h) and AT8 (c,e,f). Gallyas-Braak silver staining was used in (d). Nuclei were counterstained. Scale bars: 50 μm (in a, for a-d), 500 μm (in e, for e,g), 100 μm (in f, for f,h). Panels (e) and (f) show the same section from occipital cortex at different magnifications.

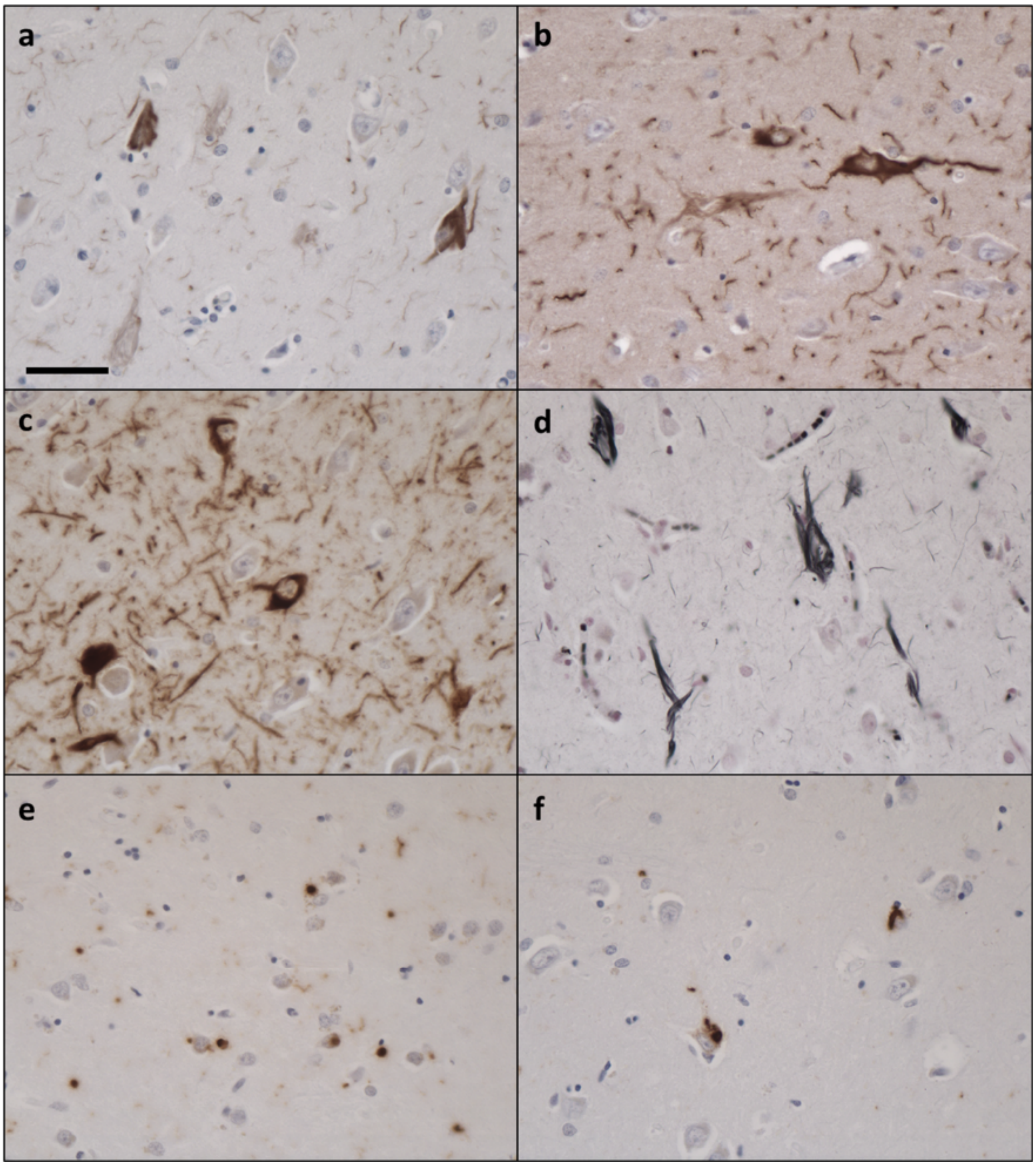

Supplemental figure 8

Tau staining of hippocampal formation from PART case 2

Immunohistochemical staining of sections from hippocampal formation (a-d) using anti-tau antibodies RD3 (a), anti-4R (b) and AT8 (c). Gallyas-Braak silver staining was used in (d). Putamen (g) and amygdala (h) were stained for TDP-43. Scale bar: 50 μm (in a, for a-h).

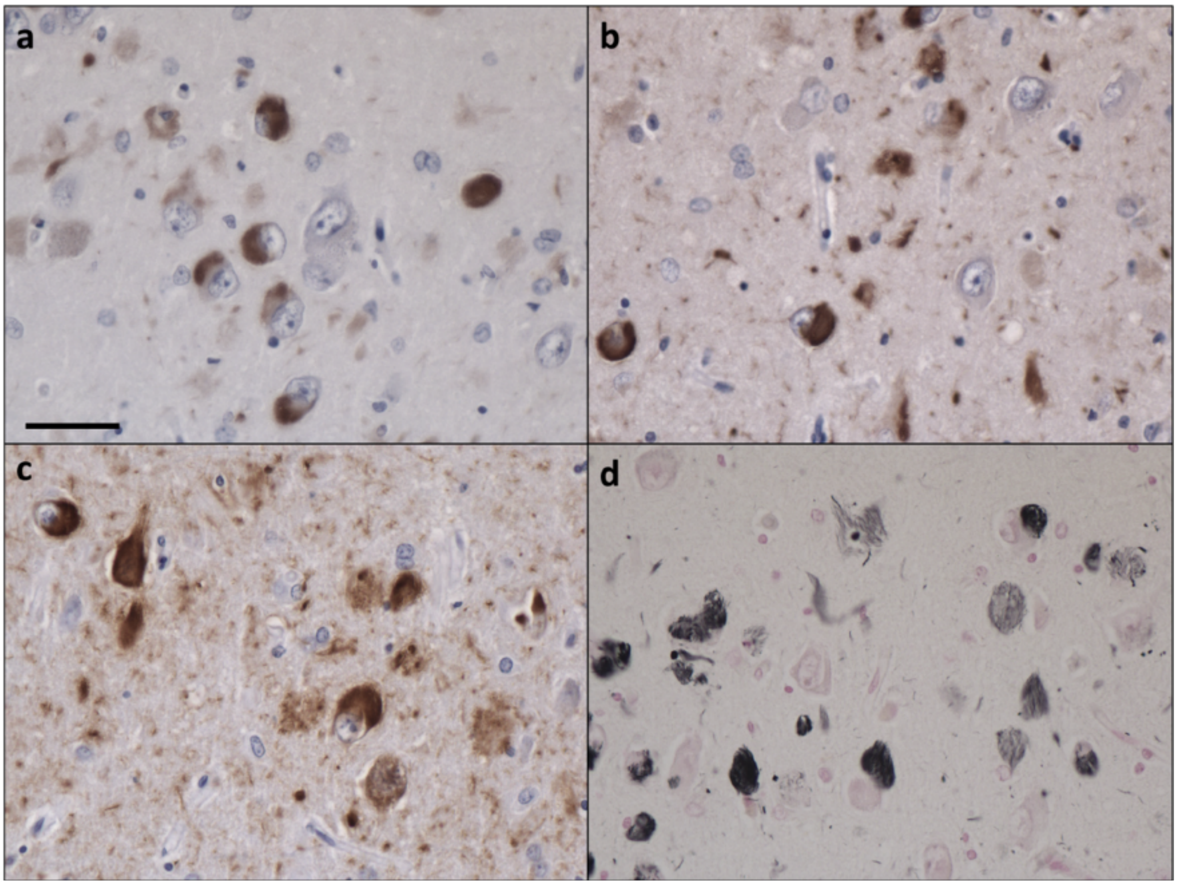

Supplemental figure 9

Tau staining of hippocampal formation from PART case 3

Immunohistochemical staining of sections from hippocampal formation (a-d) using anti-tau antibodies RD3 (a), anti-4R (b) and AT8 (c). Gallyas-Braak silver staining was used in (d). Scale bars: 50 μm (in a, for a-d).

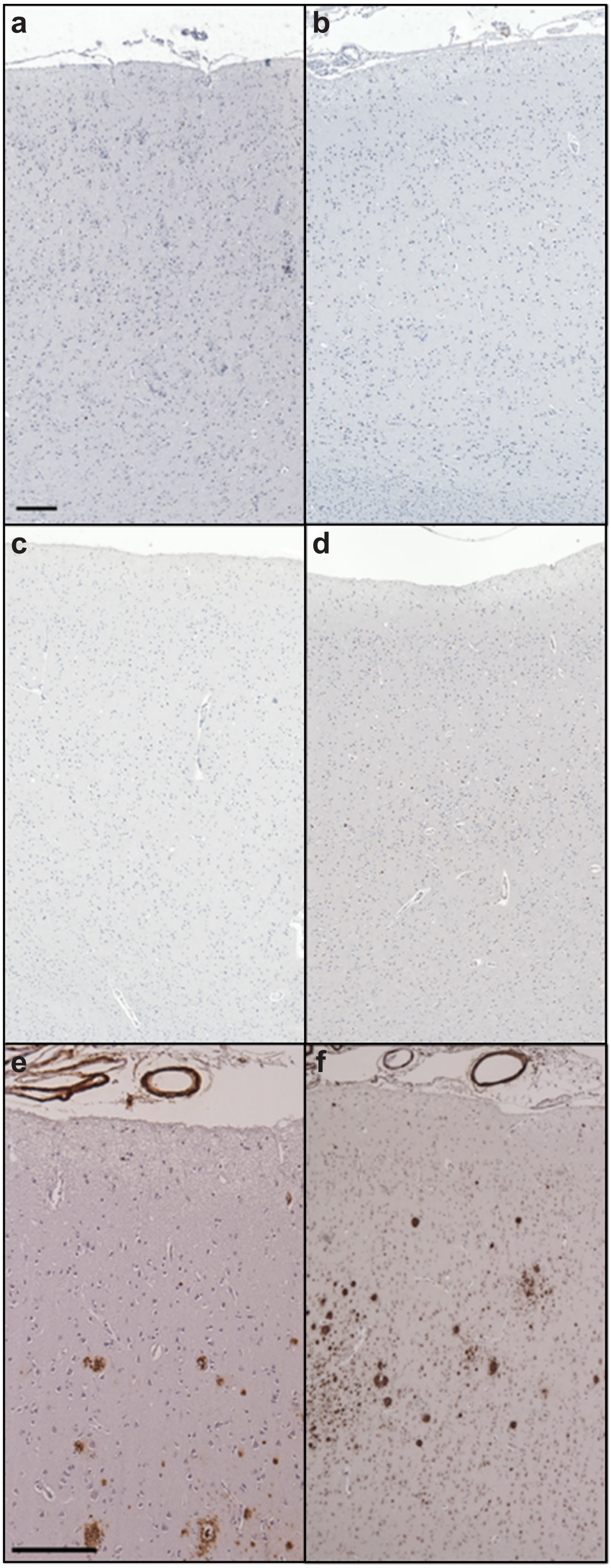
a
b
c
d
e
f

Supplemental figure 10

Aβ staining of cerebral cortex from PART cases 1-3

Immunohistochemical staining of sections from frontal (a,c,e) and temporal (b,d,f) cerebral cortices of PART case 1 (a,b), PART case 2 (c,d) and PART case 3 (e,f) using anti-Aβ antibody 4G8. Scale bars: 200 μm (in a, for a-d, and in e, for e,f).
